# Reconstruction of Tumor-Induced Pelvic Defects With Customized, Three-Dimensional Printed Prostheses

**DOI:** 10.3389/fonc.2022.935059

**Published:** 2022-06-30

**Authors:** Shenglin Xu, Zehao Guo, Qiling Shen, Yongjun Peng, Jian Li, Sheng Li, Peng He, Zheng Jiang, Yukang Que, Kun Cao, Bo Hu, Yong Hu

**Affiliations:** Department of Orthopedics, The First Affiliated Hospital of Anhui Medical University, Hefei, China

**Keywords:** endoprosthesis, porous, 3D printing, pelvic tumor, hemipelvic reconstruction

## Abstract

**Background:**

Reconstruction of pelvis girdle stability after tumor-induced hemipelvectomy remains challenging. We surgically treated 13 patients with custom-made, three-dimensional printed hemipelvic prostheses. We aim to identify the preliminary outcomes for patients who have been managed with more mixed regions of prosthetic pelvic reconstruction and the feasibility of two reconstructive systems.

**Methods:**

Seven male patients and 6 female patients treated at our center between January 2019 and May 2021 were included. There were 11 primary sarcomas and 2 solitary bone metastases. After en bloc tumor resection, two types of personalized, three-dimensional printed prostheses were fixed to restore the stability and rebuild the load transfer. The position of the reconstructed hemipelvis was evaluated on an anteroposterior plain radiograph. The complications and outcomes were traced. One amputation specimen was discovered through histological analysis of the porous structure.

**Results:**

The operative duration was 467 ± 144 min, and the blood loss was 3,119 ± 662 ml. During a follow-up of 22.4 ± 8.5 months, two patients had delayed wound healing and one had a second-stage flap transfer. One patient with osteosarcoma died of pulmonary metastasis 27 months after surgery. Two patients with marginal resection suffered from local recurrence and had extra surgeries. One patient had traumatic hip dislocation 2 months after surgery and manipulative reduction was performed. The acetabular inclination of the affected side was 42.2 ± 4.3°, compared with 42.1 ± 3.9° on the contralateral side. The horizontal distance between the center of the femoral head and the middle vertical line was 10.4 ± 0.6 cm, while the reconstructed side was 9.8 ± 0.8 cm. No significant difference in acetabular position after surgery was found (*p* > 0.05). The amputation specimen harvested from one patient with local recurrence demonstrated bone and soft tissue ingrowth within the three-dimensional printed trabecular structure. Walking ability was preserved in all patients who are still alive and no prosthesis-related complications occurred. The MSTS score was 22.0 ± 3.7.

**Conclusions:**

Both types of custom-made, three-dimensional printed prostheses manifested excellent precision, mechanical stability, and promising functional rehabilitation. The porous structure exhibited favorable histocompatibility to facilitate the ingrowth of bone and soft tissue.

## Introduction

Pelvis girdle accounts for 10% to 15% of primary malignant bone tumors and ranks third among bone metastasis sites (1). The initial procedure choice of external hemipelvectomy, the so-called hindquarter amputation, may bring considerable physical and psychological disability and reduce the quality of life dramatically. With the advent of better imaging and more advanced surgical techniques, limb-sparing surgeries have been proposed for patients suffering from primary pelvic malignancies and some metastatic lesions (2, 3). Palliative surgery such as iliofemoral or ischiofemoral arthrodesis (4), although better than amputation, is not accepted nowadays. Various methods have been applied for pelvic reconstruction after tumor resection with adequate margins, such as osteoarticular allograft/autograft (5) and metallic endoprostheses reconstruction using the saddle prosthesis (6), pedestal cup prosthesis (7, 8), and customized prosthesis (9). Since only anatomical and cosmetic reconstruction was concerned in the early stage, the characteristics of load transfer and the osseointegration potential were ignored. Consequently, due to the high incidence of complications including deep infection, prosthesis breakage, and recurrent dislocation, amputation is inevitable in some patients (10).

A porous structure can effectively reduce the elastic modulus of the implants to prevent the occurrence of stress shielding, and offer space for angiogenesis and bone regeneration, which is conducive to tissue repair, early rehabilitation, and biological fixation (11). Currently, additive manufacturing, also known as three-dimensional printing (3D) technology, featuring rapid manufacture and precision, has led to changes in the medical field, especially in orthopedics and plastic surgery. 3D printing technique addresses some potential shortcomings of prostheses using conventional methods, such as uncontrollable mechanical properties and pore parameters. Computer-aided designed implants tailored by 3D printing with porous titanium scaffolds can provide precise contour of tumor-induced bone defects and modified architectural characteristics, thus reducing the long-term mechanical complications and promoting better function of limbs (12).

In this study, thirteen patients suffering from primary or metastatic peri-acetabular tumors underwent resection of the lesion and reconstruction with personalized, 3D-printed prostheses. This study aimed to (1) explore limb function after prosthesis implantation; (2) identify outcomes associated with the prostheses and surgery; and (3) access the feasibility of a novel pedicle screw–rod prosthesis system to achieve solid lumbopelvic fixation.

## Materials and Methods

### Patients

Between January 2019 and May 2021, thirteen patients with pelvic malignant tumors treated at our center were enrolled in this study (**Table 1**). Reconstruction with customized, 3D-printed endoprosthesis was indicated if the following criteria were met: (1) pathological diagnosis of chondrosarcoma, or primary sarcoma with promising neoadjuvant chemotherapy sensitivity, or solitary metastatic lesion; (2) life expectancy longer than 6 months; (3) lesions not involving the sacral nerve or sciatic nerve; and (4) being willing to accept the 3D-printed prosthesis rather than modular implant. The cohort consisted of 7 male patients and 6 female patients with an age of 54.7 ± 11 years. According to the classification proposed by Enneking et al. (13), eight patients had region I + II + III lesions, four had region I + II lesions, and one had a tumor located in region II + III.

**Table 1:** Demographics, complications, and outcomes of the patients.

Patient number	Gender	Age (years)	Pathological diagnoses	Resection classification	Surgery duration (min)	Blood loss (ml)	Additional surgery	Complications	Follow-up (months)	Status	MSTS-93
1	Female	52	CS	II + III	245	2,764	Amputation		11	NED	25
2	Female	52	CS	I + II	345	1,973			23	AWD	26
3	Male	57	CS	I + II	410	2,857			36	NED	25
4	Male	69	CS	I + II	375	3,363		Dislocation	20	NED	21
5	Male	43	OS	I + II	480	2,932			28	AWD	22
6	Male	75	OS	I + II + III	660	3,923	Cystostomy + DJUS implantation		28	NED	16
7	Female	54	Renal cell cancer	I + II + III	320	2,907			37	AWD	26
8	Male	66	Prostate cancer	I + II + III	300	2,370			12	AWD	26
9	Female	55	CS	I + II + III	510	3,215		DWH	14	NED	16
10	Male	53	CS	I + II + III	620	3,333	Prosthesis-preserving tumor resection		14	NED	23
11	Female	53	CS	I + II + III	600	3,098			20	NED	17
12	Female	50	CS	I + II + III	660	4,657		DWH	21	NED	22
13	Male	32	OS	I + II + III	540	3,150			27	DOD	22

CS, chondrosarcoma; OS, osteosarcoma; DJUS, double-J ureteral stent; DWH, delayed wound healing; NED, no evidence of disease; AWD, alive with disease; DOD, died of disease.

Initial diagnosis was made by needle biopsy and verified by postoperative specimen. Among all the patients, there were 8 cases of chondrosarcoma, 3 instances of osteosarcoma, and 2 had pelvic oligometastases from the kidney and prostate.

Functional evaluation of the affected limb was determined by Musculoskeletal Tumor Society (MSTS) scores (14) at the latest follow-up. Pelvic radiographs after surgery were evaluated to compare acetabular inclination and position of the hip rotation center using RadiAnt DICOM Viewer software (Version 2020.2, Medixant, Poland). Inclination of the acetabular cup was measured as the angle between the borders of the cup and the teardrop line (3). The height of rotation center was defined as the vertical distance between the femoral head and the line connection of bilateral ischial tuberosity. The measurement was carried out by two experienced orthopedic surgeons, and the average value was used as the final result for statistical analysis. Osteointegration and soft tissue ingrowth were evaluated in an amputation specimen.

### Prosthesis Design and Fabrication

**Figure 1:**
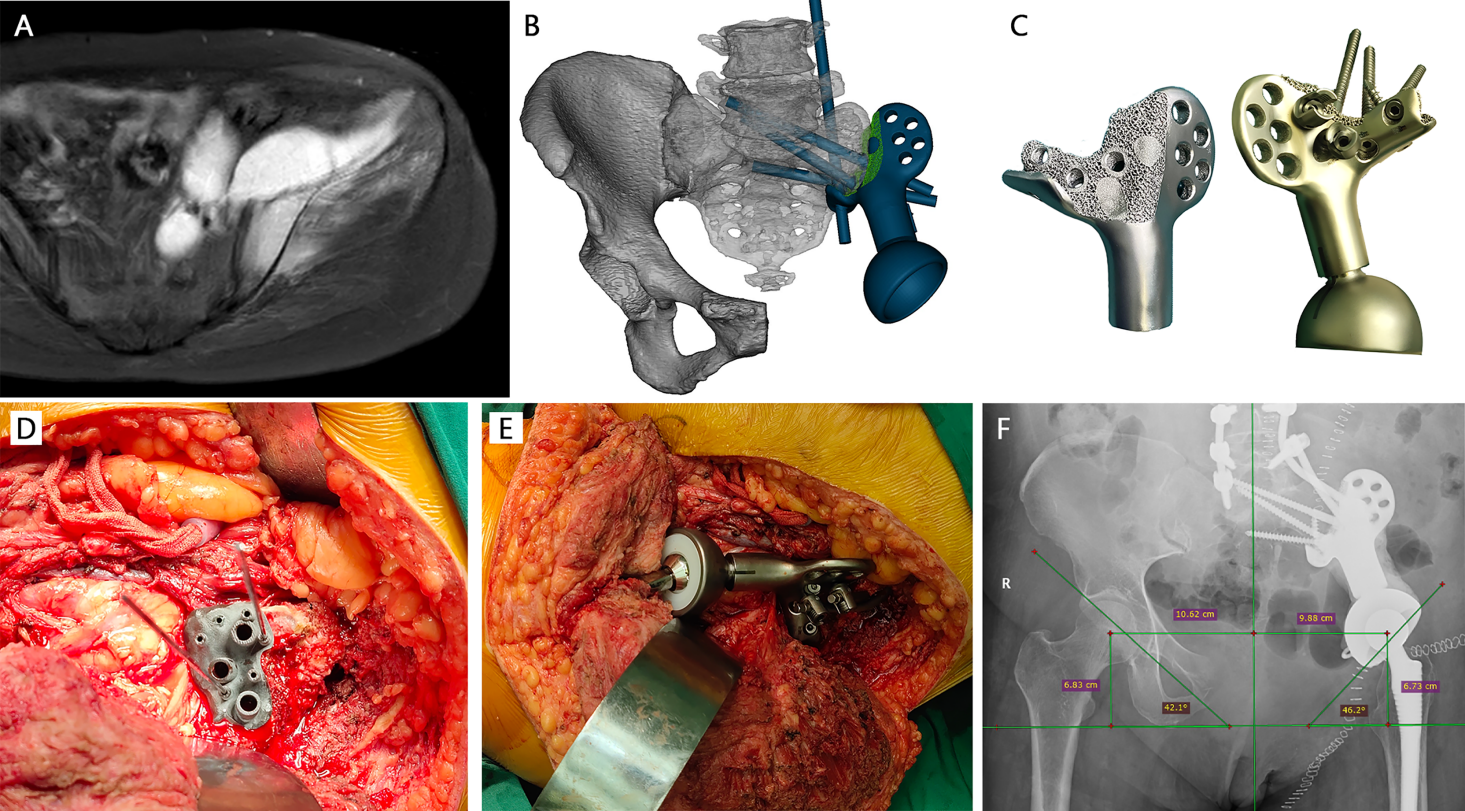
A 53-year-old female patient with chondrosarcoma. **(A)** Preoperative MRI showed the invasion of the sacroiliac joint. **(B)** The design of Type A prosthesis (green part: porous structure; blue part: solid structure). **(C)** The 3D-printed prosthesis with lattice bone contact surface. **(D)** Intraoperative installation of patient-specific drill guide at the auricular surface. **(E)** Installation of 3D-printed prosthesis. **(F)** Immediate x-ray after surgery showed that the prosthesis was fitted precisely.

**Figure 2:**
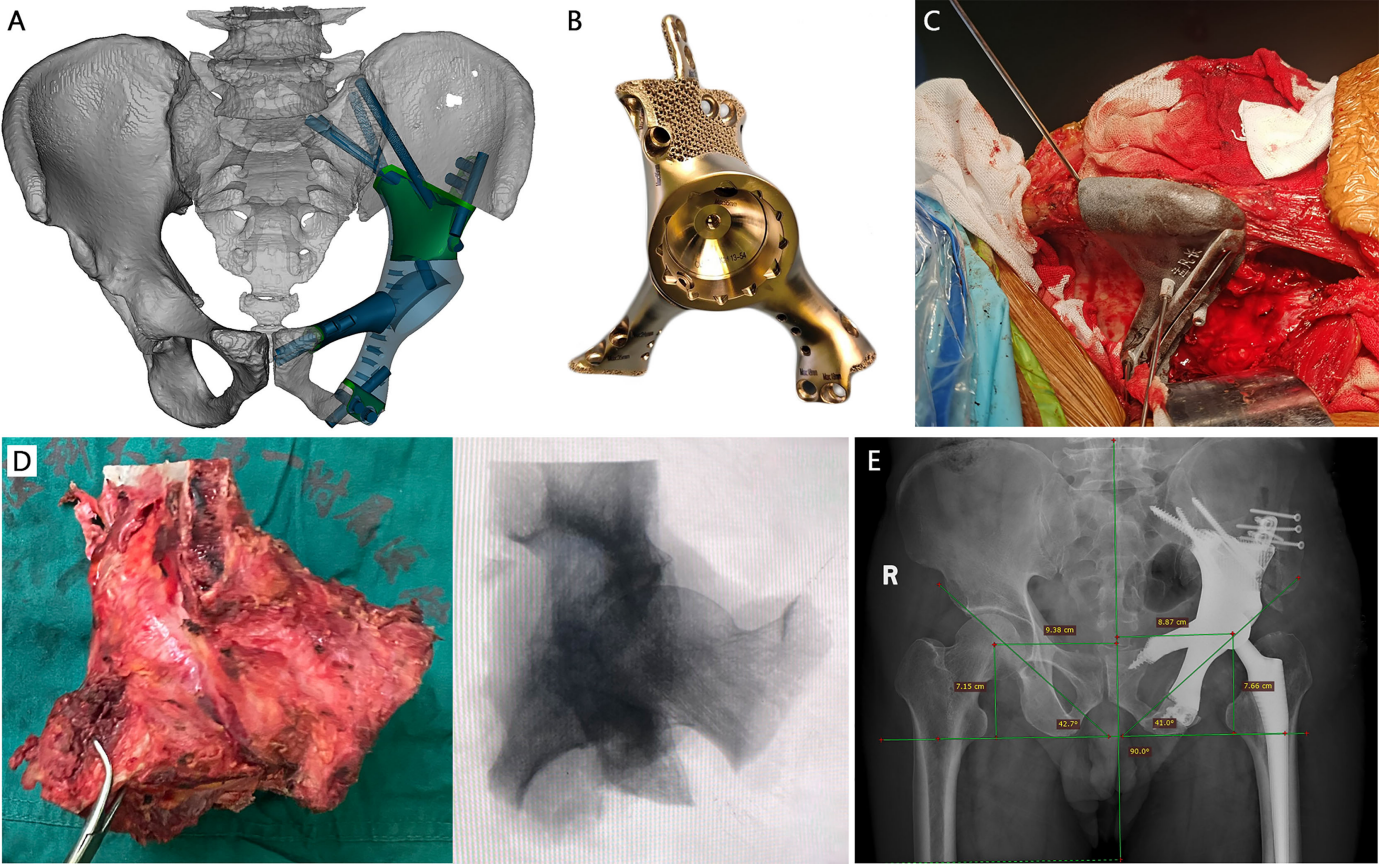
A 66-year-old male patient with metastatic prostate cancer. **(A)** The design of type B prosthesis after partial type I + II + III resection (green part: porous structure; blue part: solid structure). **(B)** The real picture of the implant. **(C)** Intraoperative installation of patient-specific osteotomy guide on the iliac crest. **(D)** Extra-articular resection of the tumor. **(E)** Postoperative x-ray manifested promising accuracy of the prosthesis.

According to the extent of residual ilium, two main types of prostheses with streamline configuration were designed. Type A reconstruction stemmed from the concept of a metallic prosthesis that could connect spine and ipsilateral lower extremity to rebuild the load transfer through a rod-screw system (**Figure 1**). The joint area between the prosthesis and the subchondral bone of the auricular surface was porous, which was supposed to achieve long-term stability after osseointegration. Sacral tray was incorporated to be fully fitting with the ventral sacrum surface to mitigate the shear force imposed on the screws. One screw, almost perpendicular, was designed to insert through the bottom of the sacral tray up to the sacral wing. The length and trajectory of the polyaxial screws were designed in advance to avoid penetrating the sacral canal and the anterior neurovascular plexus. The screws can be either fixed to the sacroiliac joint and vertebra or designed to be part of the pedicle screw–rod system. Five patients (patients 9–13) with complete type I + II + III resection were reconstructed in this manner. The principle of type B endoprosthesis was to restore the peri-acetabular region (**Figure 2**). Extracortical plate and screws were designed to act as the main anchorage tool of the implant to the remaining iliac wing. Distribution of screws was radial for better stress dispersion. Reconnection of the ischiopubic ramus was performed in patients whose pubic symphysis could be preserved. Acetabular cup orientation was pre-measured for anteversion and inclination. 3D-printed PSGs, including cutting guides and pre-drilled guides, were designed according to the natural bony structures and then fabricated from nylon powder using a selective laser sintering technique. After anatomic placement, the PSGs could be secured by 2.0-mm K-wires *via* drill sleeves. A platform that confined the oscillating saw or provided the drill trajectory was incorporated.

Digital imaging and communications in medicine (DICOM)-based CT scans for the pelvic region at 0.625-mm slice thickness were imported into Mimics software (16.0, Materialise Inc., Leuven, Belgium) to reversely construct the 3D model. The proceeding images of contralateral hemipelvis were used to create a mirror model for prosthetic design. After debugging, the prosthesis was manufactured by a 3D printing technique (Chun Li Zheng Da Medical Instruments Corporation, Ltd., Beijing, China) using titanium alloy (Ti6Al4V). The electron beam melting (EBM) technique, which is based on the powder bed fusion technique to manufacture trabecular pores, was used in fabrication with solid subjects and regions of cancellous surfaces to promote tissue ingrowth according to a computer-aided design (CAD) model. The metallic alloy powders were melted by the energy emission from the electron beam of a tungsten filament and printed layer by layer. The surface of the product was then subjected to postprocessing and sterilization before clinical use. Pore characteristics were heterogeneous, with a mean porosity of 70% and a pore diameter of 600 μm.

It took approximately 2 weeks for the design and manufacture of each personalized 3D-printed prosthesis and PSG.

### Surgical Procedure

All operations were performed by the same senior surgeon (YH). For implantation of type A prosthesis, the surgeries consisted of two stages with combined posterior and lateral approaches. In the remaining cases, only stage II was taken. The range of soft tissue mass was carefully separated according to preoperative MRI images and a wide resection margin was pursued.

Stage I: posterior approach

With patients in the prone position, a posterior median incision was made. Four transpedicle screws were placed into the bilateral L4 and L5 pedicles, and one S1 pedicle screw was contralaterally introduced. An auxiliary incision was adopted arcing laterally from the level of S2 to explore the protruding soft tissue mass and ligate the perfusion arteries. Under the premise of ensuring a clear surgical margin, parts of the gluteus medius and minimus were retained. The nutrient vessels that stemmed from the superior gluteal artery were preserved unless violated by tumors.

Stage II: lateral approach

The patients were repositioned in a lateral decubitus position on the contralateral side. An incision combined an extended ilioinguinal incision and a vertical incision toward the greater trochanter was applied. Continued from the arc-shaped incision located posteriorly if stage I was adopted, the incision was made through the anterior superior iliac spine and lateral to the pubic tubercle, ending at the medial aspect of the ipsilateral thigh. The common iliac artery was exposed for temporary occlusion to prevent severe bleeding followed by separation along the extraperitoneal space. Based on the size of the tumor, the extent of iliopsoas resection varied. If the osteotomy through the ischial ramus was planned, the adductor muscles were separated and the tendons attached to the ischium were resected.

Sacroiliac joint was disarticulated anteriorly and posteriorly concurrently in five patients designed with type A prosthesis implantation. The sciatic nerve and presacral plexus were avoided. The auricular cartilage was then exfoliated using curettes and rongeurs, removing all cartilage back to the subchondral bone. Patient-specific guide, fitting on the native bony structure, was firmly seated and stabilized with K-wires to execute pre-planned osteotomy and predrill the screw trajectories for implant fixation. The type A prosthesis was compactly fixed with the sacral tray fully fitting to the anterior edge of the sacrum and rods were bilaterally connected to achieve lumbopelvic fixation (**Figures 1D, E**). After two PSG-aided osteotomies at predetermined positions (**Figure 2C**), the portion of the pelvis containing the tumor was removed en bloc and the type B prosthesis was attached to the residual ilium. The acetabular component was press-fit and augmented with antibiotics-laden bone cement, followed by installation of a femoral implant and reduction of the artificial hip joint. Pulsatile lavage with large amounts of saline solution was used to rinse the surgical area.

For soft tissue reconstruction in patients with type A prosthesis implantation, the rectus femoris and the biceps femoris were reattached to the suturing hole of bone cement with a nonabsorbable suture. The residual glutei and tensor fasciatus were sutured to the body of the prosthesis through reconstructive holes reserved and the abdominal muscles. The attachment of adductor muscle was reconstructed to the rectus abdominis muscle. The wound was closed in layers. R0 resection was achieved in all patients.

### Postoperative Management

During the initial 2 weeks, patients were constrained to a neutral lower extremity position with the hip flexing slightly to reduce the tension of incisions. Range of motion was restricted to external rotation and flexion less than 90° for 4 weeks to prevent hip dislocation. Drainage tube placed in surgical area under negative pressure was removed when the volume of drainage was less than 50 ml in 24 h. Low-molecular-weight heparin calcium with a dose of 4,000 IU/day was administered for prophylactic anti-deep vein thrombosis. A third-generation cephalosporin was given for 14 days, followed by oral combination of levofloxacin and rifampicin for 1 month. After 8 to 10 weeks of bed rest to allow scar tissue formation and construction stabilization, patients were encouraged to begin ambulation and progressive weight bearing

### Statistical Analysis

SPSS software version 25.0 (IBM, Armonk, New York) was used for all statistical analysis, and the data were expressed as mean ± standard deviation (SD). Positions of native and reconstructed sides were compared with paired *t*-test. A *p*-value < 0.05 was considered statistically significant.

## Results

### Operational Outcomes

The operative duration was 467 ± 144 min and the blood loss was 3,119 ± 662 ml. One patient had additional urinary tract reconstruction at the same time due to tumor invasion (**Table 1**). Wide surgical margins were accomplished in eleven patients, and the two remaining patients had a marginal margin. All patients had intraoperative blood transfusions. The amount of red blood cell transfusion was 8 ± 3 units, and three patients were transferred to the intensive care unit (ICU) for 3 days before returning to the ward.

### Perioperative Complications

Two patients had wound healing disturbance at a total of three sites, including one at the posterior curved incision and two at the crossing site of the T-shaped incision. One patient underwent an operation consisting of three cycles of irrigation and debridement of devitalized tissue. Incision was left open with vacuum-assisted closure (VAC) and suture was attempted 1 week later, but failed. A second-stage flap transfer was then performed. The other patient underwent simple debridement and closure procedure. The wound healed 2 weeks later. One patient suffered from traumatic hip dislocation 2 months after surgery and manipulative reduction was performed. No signs of aseptic screw loosening or deep infection were observed in the study.

### Imaging and Functional Outcomes

Chest CT was taken every 6 months and contrast-enhanced MRI of the pelvis was taken every 1 year. The patient underwent evaluations including physical examinations and plain radiography before discharge and 1, 3, 6, and 12 months after surgery and every 1 year thereafter. The functional assessment ended before a second surgery such as amputation, which might severely impair limb function permanently. According to postoperative plain radiography, the acetabular inclination of the affected side was 42.2 ± 4.3°, compared with 42.1 ± 3.9° on the contralateral side (*p* = 0.887). The horizontal distance between the center of the native femoral head and the middle vertical line was 10.4 ± 0.6 cm, while the reconstructed side was 9.8 ± 0.8 cm (*p* = 0.375). The height of the rotation center was also nearly the same on both sides after reconstruction (7.4 ± 0.9 cm vs. 7.9 ± 0.8 cm) and no statistical significance was found (*p* = 0.715). Detailed measurement data were presented in the **Supplementary Materials**. The walking ability was preserved in all patients. The MSTS-93 score was 22.0 ± 3.7 at the last follow-up, indicating a promising function recovery.

### Oncological Outcomes

One patient with osteosarcoma died of pulmonary metastasis 27 months after surgery while others were alive at the latest follow-up. Two patients with marginal resection experienced local recurrence. One received a palliative prosthesis-preserving surgery while the other underwent a hindquarter amputation. The disease-free interval was 8 and 11 months each. Two patients developed multiple lung metastases 9 months and 14 months respectively after surgery, and were treated with chemotherapy, target therapy and immune checkpoint inhibitors. The size and number of metastases did not progress further. For the two patients diagnosed with metastatic malignancies, the primary tumor showed no significant progression during the period (**Table 1**).

### Histological Outcomes

**Figure 3:**
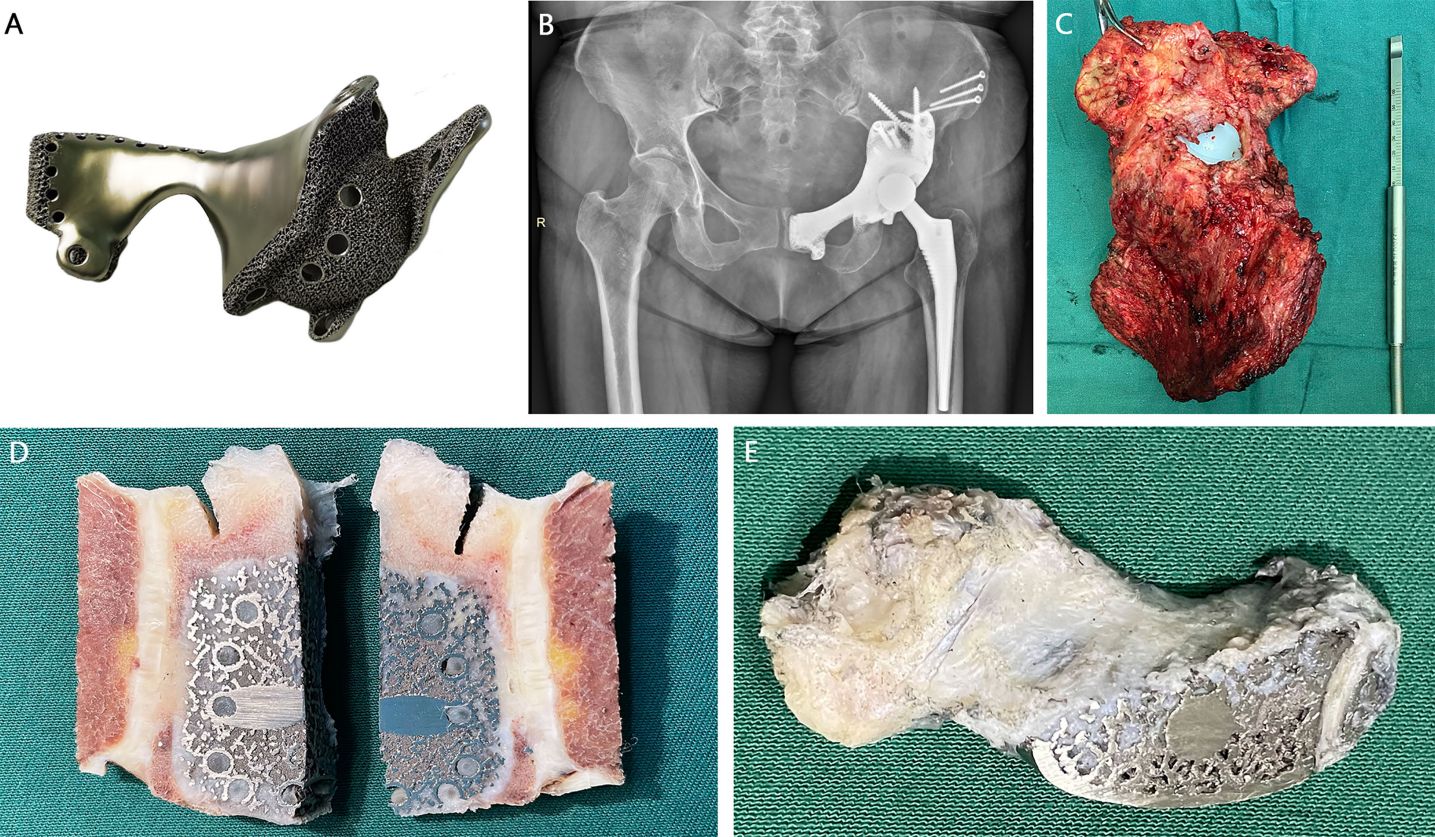
A 52-year-old female patient with recurrent chondrosarcoma 11 months after prosthetic implantation. **(A)** Type B prosthesis after type II + III resection. **(B)** Postoperative x-ray showed excellent shape compatibility. **(C)** The gross specimen of the affected hemipelvis. **(D)** The cross section showed the osseointegration ability of the porous structure. **(E)** Fibrous tissue could extend to the 3D-printed macro pores.

Hindquarter amputation specimen harvested from patient 1 due to local recurrence was used for histological analysis (**Figure 3**). The portion of the prosthesis adjacent to the pubic symphysis was cut for cross-sectional analysis. Osseointegration was found at the bone/prosthesis interface, with discernable bone ingrowth in the porous structure (**Figure 3D**). Moreover, the contact site between the prosthesis and the soft tissue was covered with dense collagen fibers. The fibrous tissue could also extend to the 3D-printed macro pores (**Figure 3E**).

## Discussion

The first symptom of pelvic tumors is usually diffuse or dull pain in the lower abdomen or hip of the affected side. Only when invading the nerve plexus does it manifest as radiating pain. Palpable lump on the body surface prompts that the tumor size is already huge at diagnosis, and mostly malignant pelvis-derived tumors, for example, chondrosarcoma in adults, and osteosarcoma and Ewing sarcoma in adolescents (15), making treatment difficult for orthopedic oncology surgeons. With lesions involving the acetabulum area, the type II resection is inevitable. Either biological methods or endoprosthetic options are attempted to restore force transmission and weight bearing along the anatomic axis. Although allografting, autografting, and prosthetic replacement are acceptable reconstructive techniques, an individualized defect shape, excessively high rates of deep infection, and mechanical failure could restrict the application. Considering the several advantages of the 3D-printed prosthesis, such as high shape compatibility, osteointegration capacity of the porous surface, and promising mechanical strength of the material (16), we performed reconstruction using customized, 3D-printed prostheses in our patients. Postoperative function was satisfactory and complications that occurred were acceptable and manageable.

There is no unanimous opinion regarding the most appropriate method of reconstruction using prostheses after extensive hemipelvic resections. Saddle prostheses or ice cream cone prostheses require the upper part of the iliac wing being left in place to avoid superior migration of the femoral head rotation center, which may limit the use in lesions involving the ilium and sacroiliac joint (7, 17). In contrast, the computer-aided designed, custom-made hemipelvic prostheses are specifically modeled to perfectly match bony defects. Implantation of customized prostheses for pelvic reconstruction after tumor resection was not associated with a reduced rate of complications but facilitated functional improvement (10). The stability of prostheses fabricated by conventional manufacturing techniques depended mostly on mechanical fixation, resulting in poor osseointegration and decreased prosthesis survival rate with time. Though possessing better bone ingrowth capacity, the inherent poor fit of the anchor part compromised for high adjustability could not be avoided in modular implants with modified 3D-printed bone contact surface (18).

The complete defect of the iliac wing cannot provide suitable anchorage for most prostheses being extensively used. The nearly vertical sacroiliac joint surface produces huge shear stress, which makes loosening inevitable. The screw–rod system combined with modular hemipelvic prosthesis was highlighted after resection of periacetabular tumor invading the sacroiliac joint (3). The pedicle screws were placed through both the pedicle and lateral aspect of the L4 and L5 vertebra body. Specialized rods were then used to connect the screws and pelvic endoprosthesis which integrated spine and prosthesis. No mechanical failures, such as screw breakage and prosthesis displacement happened. Nonetheless, it was then ascertained that the application of lateral lumbar vertebral body screws remained as an independent risk factor of postoperative neuralgia (19).

For prostheses made of dense-type titanium alloy, with hydroxyapatite coated surface being the most widely used method to pursue biological fixation, bone tissue can only extend to the surface area and not the interior of the implants, and long-term stability is difficult to guarantee in the pelvic region where mechanical internal fixation carries enormous shear stress due to complex biomechanics. Porous structures, acting as weight-bearing scaffolds with high pore connectivity for bone ingrowth and osseointegration, have been used in prosthesis manufacturing. Currently, custom-made prostheses manufactured by conventional techniques are as precise, if not more, than those fabricated using additive manufacturing. However, traditional technologies such as foam fabrication, plasma spraying, and powder sintering were restricted by the inherent inability to control the porosity and high possibility of pore blockage (20). Benefiting from the in-depth development of 3D printing technology, the macro pores can be controlled according to the CAD model to modify mechanical properties and biological performance. Moreover, porosity allows for a reduction in the elastic modulus of metal, thus potentially alleviating the effects of stress shielding.

3D-printed implants could be manufactured with special reference to bone defects possessing complex spatial geometry. Studies on the clinical applications of 3D printing technology in hemipelvic prosthetic reconstruction have mostly been case reports. In 2015, Wong et al. (21) and Fan et al. (22) reported one case of 3D-printed prosthesis for sarcoma-induced pelvic defects, respectively. The region that needed reconstruction was relatively limited, with one occupying approximately 40% of the acetabulum, and the other the ilium and upper rim of the acetabulum. Since then, multiple center-derived utilization of 3D-printed pelvic prostheses has emerged (**Table 2**). Desirable function outcome and complication rate were retrieved in relatively small cohorts and short follow-up periods, with wound complications being the most frequent and no prosthetic failure reported, which concurred with our study. Nonetheless, the approach to prosthesis design differed. In some instances, the attention to biomechanical characteristics was neglected, and the reconstructed area was limited.

**Table 2:** Comparison of the 3D-printed, custom-made hemipelvic prosthetic reconstruction studies.

Year	Author	Resection classification	Case number	Follow-up (months)	MSTS-93	Complications				
						DWH		IF	DL	HT
Current study		II + III I + II I + II + III	1 4 8	22.4 (11–37)	22.0 (16–26)	2			1	
2017	Wang (23)	Including II	11	15.5 (6–44)	19.2 (13–25)	1			2	
2020	Wang (24)	I + II I + II + III	3 10	27.3 (24–31)	Median 23 (15–27)	2				
2021	Peng (25)	I + II II + III I + II + III	2 1 3	19.8 (15–26)	19.8 (15–26)	None				
2021	Xu (26)	I II	5 5	11.9 (6–24)	23.8	NA				
2021	Pu (27)	I	12	Median 21	24.1	1				

DWH, delayed wound healing; IF, infection; DL, dislocation; HT, hematoma; NA, not available.

The bone defect due to hemipelvectomy leads to disconnection between spine and ipsilateral lower extremity. Compared with iliosacral screw fixation, restoration of the continuity and hip function by lumbopelvic fixation could allow early ambulation and thus reduce pulmonary complications caused by long-term bed rest in patients with posterior pelvic ring disruption (28). The design of type A prosthesis stemmed from the concept of a porous metallic prosthesis that could connect the lumbosacral spine and hip joint, and rebuild the structure of loading transfer through a rod–pedicle screw system together with major screws fixed to perform sacroiliac arthrodesis.

In traditional modular or customized prostheses, the long axis of screw placement was often almost parallel to the plane of the pelvic ring, which causes significant shear force perpendicular to the fixed screws, greatly increasing the risk of implant loosening and breakage (29, 30). In order to improve this defect, the structure of sacral tray was incorporated according to the anteversion angle of the sacrum, accompanied by improved load-bearing capacity and diminished shear force. Provisional rigid fixation and compression are prerequisites for osteogenesis at the interface. The pedicle–rod system was also proved to significantly increase the stiffness of the connection between the prosthesis and the residual sacrum. It is worth noting that the addition of the posterior lumbosacral procedure in selected patients increases the operation time, surgical trauma, and blood loss. The firm internal fixation will weaken the stress stimulation at the bone contact surface, which is not conducive to osteogenesis, and this could be ameliorated by the porous interface. Moreover, displacement and stress bilaterally transferred along the iliofemoral load bearing axis were distributed more evenly (31). Bone–prosthesis fusion implies a firm, direct, and lasting connection. Titanium alloy 3D printing technology based on EBM processes interconnected porous structures, providing the strength required while facilitating bone formation deep within the pores (12), which has been proved by micro-CT, T-SMART scanning, and amputation specimens (3, 32). Contrary to the homogeneous pore structure previously reported, which would undesirably increase the interface stress, the pores employed in this study were irregular, i.e., pore sizes varied in different regions, and the diameters in the same area formed a gradient to mimic the native cancellous bone (33). The metallic wires forming the edge of pores provide a scaffold for cell adhesion. The mean pore size of 600 mm was a trade-off between high higher connectivity and load-bearing capacity, which ensures the maximum osteogenic effect while meeting the required mechanical strength. The mesh-like newborn bone firmly secured the prosthesis to achieve biological reconstruction, as can be observed in the longitudinal section of the specimen. Nevertheless, early, excessive weight-bearing should be avoided.

The concept of mechanical restoration instead of anatomical restoration has been accepted. The custom-made prostheses in this study were designed according to the mirror image of the contralateral hemipelvis, with complete filling of the native bone structure, while omitting the iliac wings and iliac crest, thus resulting in a much smaller volume, making it easier for flap coverage. The anteversion and inclination of the acetabulum were pre-measured and determined, requiring no additional adjustment during the operation and ensuring complete symmetry of the height, angle, and distance from the midline on both sides. Comparing the reconstructed side with the contralateral counterpart, there were no significant differences in aspect of acetabular location and orientation. In addition to the error of manual measurement, the scraping thickness of the sacroiliac joint surface, the slightly wider PSG slots and soft tissue remaining on the surface of the bony structure where the PSGs are placed caused errors in the osteotomy position. There was no obvious breakage and displacement of the prostheses on the postoperative x-rays. However, only high-resolution images in DICOM format were qualified for measurement, so it is difficult to track the subtle dynamic changes in prostheses position during the follow-up, which is where our research needs to be improved.

Although limb-sparing surgery seems superior to amputation for maintaining body integrity and limb function, it should not jeopardize the goal of adequate tumor resection (34). For a complete type I + II + III resection, no PSG-aided intraoperative osteotomy was executed. Otherwise, the application of a 3D-printed osteotomy guide was employed to make the cutting plane more accurate as planned (35). The correct placement of the sacral screws is crucial. Through CT image-based preoperative reconstruction, the direction and length of the screws are determined to avoid penetration of the sacral canal and presacral plexus. 3D-printed pre-drilled guides are used to help transpose during operation. In our study, there were no patients with complications related to sacral nerve damage. However, the margin of soft tissue could only be identified by preoperative MRI images and the experience of the surgeon. Intralesional resection and positive margin presented by histological examination were proved to be responsible for local recurrence and poor outcome. For lesions extending to the sacrum, where adequacy of resection was hard to ensure for preservation of the sacral nerves, the rate of recurrence was higher (36).

The dynamic stability of the prosthesis depends on the preservation of the surrounding soft tissues and the degree of muscle tension. The prostheses were pre-designed with suture holes for soft tissue reattachment to reduce the risk of hip dislocation. Since the main part of the pelvic girdle stability is provided by the posterior pelvic ring, reconstruction of the anterior ring seems to be unnecessary. Destruction of the anterior ring generated a separating tendency of the pelvic girdle and increased the withdrawing force at the sacral fixation, which was supposed to be balanced by lumbopelvic fusion and the sacral tray. Even if the breakage of the pubic connection plate occurred, revision surgery was not recommended for being asymptomatic in most cases and not jeopardizing subsequent function (37). In patients with traumatic pelvic ring rupture, no difference was found in quality of life and functional scores with or without the anterior ring fixation. Additionally, patients who underwent combined anterior–posterior ring fixation presented longer surgery time and operation–discharge interval (38). Zhang et al. (39) attempted to reconstruct the pubic symphysis in patients with pubis defects. Fretting wear happened eventually around the stem inserting into contralateral pubis due to the low mobility of the sacroiliac joint. Neither breakage nor loosening of the prosthesis occurred in our study, indicating that the isolated solid fixation of the posterior pelvic ring is sufficient to provide stability in the early stage, though increasing the peak stress at the iliac fixation screws in a gait cycle (40). In cases where the remaining ipsilateral pubis-ischial ramus is sufficient to attach the prosthesis, we recommend the prosthetic reconnection of region III. We infer that the stress transmitted through the metallic prosthesis can be partially relieved by the micro-motion of the pubic symphysis, thus avoiding stress concentration. The stiffness mismatch between the bone and prosthesis could be reduced *via* the introduction of the 3D-printed porous structure to facilitate force release and transfer (41). Although the internal fixation device may extend to the contralateral side, short-term stabilization is beneficial to the osteointegration of the bone–prosthesis interface to restore the integrity of the pelvic ring. Secondary osseous stability can be maintained even if the mechanical stabilization fails due to later rupture of the trans-pubic symphysis screws. However, in the case of complete resection of the region, we believe that mechanically connecting the prosthesis with a pubic rod or plate to the contralateral hemipelvis would lead to excessive concentration of shear stress and increased risk of anterior ring breakage, and the fixation of the posterior ring should be properly strengthened.

In pelvic reconstruction surgery, effective control of infection is the key to extending the life of the prosthesis and improving the prognosis. Uncontrollable deep infection leads to prosthesis removal and lengthy exposure to systemic antibiotics. High incidence of infection mainly comes down to the lengthy operation time, extensive incision, exposed blood vessels, close proximity of the intestines, and implantation of megaprostheses. Vancomycin-impregnated bone cement made of poly(methyl methacrylate) rendered a prolonged release of antibiotics while augmenting the load-bearing capacity of prosthesis (42). The wound was flushed repeatedly with a pulsing squirt gun to mitigate the risk of infection. In smooth-surfaced metallic prostheses, due to the existence of the foreign body response, implants are often sealed by dense scar, causing impaired tissue integration and vascularization. The separation of the prosthesis and the surrounding tissue creates a potential cavity while producing relative sliding, increasing the risk of deep infection. 3D-printed highly cancellous surface was reported to facilitate soft tissue ingrowth during revision surgery (43). In this study, we first discover the soft tissue infiltration in the heterogeneous porous structure. The gross appearance of the surgical specimen showed a tight fusion of soft tissue at the non-bone contact cancellous surface. The cross-section confirmed that the soft tissue could extend into the interior of the prosthesis, which is imperative for implant longevity and infection prevention.

With regard to the refractory wound complication happening in one patient, reports are quite limited. The etiology was likely to be the need to sacrifice vessels to remove such scale of the entire tumor. Skin necrosis was observed at the intersection of mutually perpendicular incisions, which has been reported in a larger scope of patients as the T-incision surgical approach was initially proposed. This region is prone to healing problems, potentially owing to inadequate blood flow and opposing skin tension (44). Debridement and regular dressing change ultimately proved to be futile, and a flap transfer was performed later in this case. Nevertheless, prophylactic flap transplantation was not recommended as a routine procedure for closure of the intersection point. Even if this minor complication happened, surgery was not required in most cases and a single incision drainage with primary closure could achieve successful healing. The adopted incision facilitated the preservation of the blood feeding of the femoral artery to the anterior flap and the superior gluteal artery to the posterior flap (45). Patients with type I + II + III resection were prone to incision complications due to the ligation of the invaded superior gluteal artery and subsequent receded perfusion of the lateral posterior flap. Thus, we proposed that the superior gluteal artery and its branches should be preserved to the greatest extent unless violated by tumors. The blood supply of the flap should be the focus of attention during and after surgery in patients with wide resection.

There are concerns about 3D-printed prostheses before widespread clinical use. In an integrated and customized design and manufacture procedure, once a certain part of the megaprosthesis fails, the entire prosthesis must be replaced, and the tight connection between the surrounding bone and soft tissues makes a reoperation more challenging. 3D-printed prostheses with modular components have been implanted in our center, and the follow-up is still in progress. The close-loop standard evaluation system for implants is lacking though patients were informed of the unknown risks. The carcinogenicity and genotoxicity associated with degradation need to keep track of the long-term outcomes, and it is imperative to establish appropriate sterilization methods and standards, especially for porous structure. In a study by McConoughey et al. (46), rough titanium alloy surfaces exhibited a higher tendency of bacterial colonization for the formation of biofilm. Improper sterilization adversely affects prostheses and can result in uncontrollable infection and a failed reconstruction. Moreover, the high cost of equipment acquisition, maintenance, and a high threshold for the use of software seriously limit its popularity.

This study had some limitations. First, the relatively short follow-up and small cohort limited our conclusions. The preliminary results reflect the advantages of the prosthesis to a certain extent considering that most surgical and implant-related complications occur in the early postoperative period (47). Follow-up is still in progress to obtain oncological outcomes and long-term complications. Multicenter collaborations are needed to recruit an adequate number of patients to form a more homogeneous group. Second, titanium alloy made it difficult to monitor the degree of osseointegration as it distorted CT and MRI images around the implant. The precise time point of osseointegration cannot be determined. Although we demonstrated the osteointegration ability of the 3D-printed macro pores through the specimen, there was a lack of effective methods to validate in other patients. Third, biomechanical analysis was not included. Finite element analysis and *in vitro* tests will further be carried out to evaluate the distribution of stress in different postures and gait circles. The role of the sacral tray and screw–rod system in prosthesis stability could also be displayed in an intuitive manner.

## Conclusion

The advent of image processing and 3D printing technology has inaugurated a new era for patient-specific reconstruction in oncological orthopedics. This study suggested novel customized, 3D-printed prostheses for the reconstruction of tumor-induced bone defects and lumbopelvic integration. The 3D-printed porous structure exhibited favorable histocompatibility, which facilitated the ingrowth of bone and soft tissue. The porous bone–prosthesis interface, perfect anatomical fitting, and continuous force transmission lead to satisfactory limb function and controllable complications. Further objective investigation of the durability and rate of long-term complications is needed before bringing this type of prosthesis into routine clinical practice.

## Data Availability Statement

The raw data supporting the conclusions of this article will be made available by the authors, without undue reservation.

## Ethics Statement

The studies involving human participants were reviewed and approved by the Ethics Committee of the First Affiliated Hospital of Anhui Medical University. The patients/participants provided their written informed consent to participate in this study.

## Author Contributions

SX, ZG, and YH designed the study and supervised the report. SX, ZJ, YQ, KC, BH, and YH performed the surgeries. ZG, QS, YP, SL, JL, and PH performed the research and analyzed the data. SX and ZG drafted the manuscript. All authors contributed to the article and approved the submitted version.

## Funding

This study was supported by the major projects of the Natural Science Foundation of Universities in Anhui Province (grant no. KJ2020ZD17) and the Program for Upgrading Basic and Clinical Collaborative Research of Anhui Medical University (grant no. 2020xkjT033).

## Conflict of Interest

The authors declare that the research was conducted in the absence of any commercial or financial relationships that could be construed as a potential conflict of interest.

## Publisher’s Note

All claims expressed in this article are solely those of the authors and do not necessarily represent those of their affiliated organizations, or those of the publisher, the editors and the reviewers. Any product that may be evaluated in this article, or claim that may be made by its manufacturer, is not guaranteed or endorsed by the publisher.
