# Supplementary material for: Reconstruction of Tumor-Induced Pelvic Defects With Customized, Three-Dimensional Printed Prostheses
Source: Front Oncol. 2022 Jun 30;12:935059. doi: 10.3389/fonc.2022.935059 (PMC9282862; doi:10.3389/fonc.2022.935059)
Supplement: Supplementary file 1 [file Table_1.docx]

Supplementary Material

# Supplementary Figures and Tables

## Supplementary Table

| Patient number | Horizontal distance (cm) | | Acetabular inclination (˚) | | Height (cm) | |
| --- | --- | --- | --- | --- | --- | --- |
|  | Native | Reconstructed | Native | Reconstructed | Native | Reconstructed |
| 1 | 10.5 | 10.6 | 42.2 | 43.0 | 6.9 | 6.8 |
| 2 | 10.2 | 11.2 | 44.9 | 45.1 | 6.4 | 5.6 |
| 3 | 9.4 | 9.8 | 42.2 | 46.2 | 8.5 | 8.1 |
| 4 | 11.6 | 10.7 | 46.3 | 45.8 | 8.0 | 8.0 |
| 5 | 7.3 | 8.4 | 33.6 | 31.7 | 8.8 | 9.2 |
| 6 | 9.7 | 8.8 | 36.9 | 34.2 | 8.5 | 8.2 |
| 7 | 10.0 | 9.4 | 41.3 | 43.2 | 7.2 | 7.7 |
| 8 | 9.5 | 8.9 | 40.5 | 40.9 | 7.1 | 7.7 |
| 9 | 10.6 | 9.7 | 48.3 | 44.7 | 6.6 | 6.8 |
| 10 | 9.9 | 9.0 | 40.4 | 41.8 | 6.9 | 6.5 |
| 11 | 10.5 | 10.0 | 42.2 | 46.2 | 8.5 | 8.1 |
| 12 | 10.6 | 10.5 | 42.0 | 43.8 | 6.9 | 7.1 |
| 13 | 10.2 | 10.4 | 42.7 | 43.5 | 7.2 | 7.7 |

**Supplementary Table 1.** Detailed measurement data of the horizontal distance, acetabular inclination and height.
